# Reconstructing DNA copy number by joint segmentation of multiple sequences

**DOI:** 10.1186/1471-2105-13-205

**Published:** 2012-08-16

**Authors:** Zhongyang Zhang, Kenneth Lange, Chiara Sabatti

**Affiliations:** 1 Department of Statistics, University of California, Los Angeles, CA, USA; 2 Department of Human Genetics, Biomathematics and Statistics, University of California, Los Angeles, CA, USA; 3 Department of Health Research and Policy and Statistics, Stanford University, Stanford, CA, USA

**Keywords:** Copy number variant, Copy number polymorphism, Fused lasso, Group fused lasso, MM algorithm

## Abstract

**Background:**

Variations in DNA copy number carry information on the modalities of genome evolution and mis-regulation of DNA replication in cancer cells. Their study can help localize tumor suppressor genes, distinguish different populations of cancerous cells, and identify genomic variations responsible for disease phenotypes. A number of different high throughput technologies can be used to identify copy number variable sites, and the literature documents multiple effective algorithms. We focus here on the specific problem of detecting regions where variation in copy number is relatively common in the sample at hand. This problem encompasses the cases of copy number polymorphisms, related samples, technical replicates, and cancerous sub-populations from the same individual.

**Results:**

We present a segmentation method named generalized fused lasso (GFL) to reconstruct copy number variant regions. GFL is based on penalized estimation and is capable of processing multiple signals jointly. Our approach is computationally very attractive and leads to sensitivity and specificity levels comparable to those of state-of-the-art specialized methodologies. We illustrate its applicability with simulated and real data sets.

**Conclusions:**

The flexibility of our framework makes it applicable to data obtained with a wide range of technology. Its versatility and speed make GFL particularly useful in the initial screening stages of large data sets.

## Background

Genomic duplications and deletions are common in cancer cells and known to play a role in tumor progression [1]. As our ability to survey the fine scale of the human genome has increased, it has become apparent that normal cells can also harbor a number of variations in copy number (CN) [2,3]. The last few years have witnessed a steady increase in our knowledge of the size and frequency of these variants [4-7] and their implications in complex diseases [8,9]. At the same time, statistical methods and algorithms have been developed to better harness the information available. At the cost of oversimplification, two different approaches have become particularly popular. One is based on the hidden Markov model (HMM) machinery and explicitly aims to reconstruct the unobservable discrete DNA copy number; the other, which we will generically call “segmentation”, aims at identifying portions of the genome that have constant copy number, without specifically reconstructing it.

The HMM approach takes advantage of the implicitly discrete nature of the copy number process (both when a finite number of states is assumed and when, as in some implementations, less parametric approaches are adopted); furthermore, by careful modeling of the emission probabilities, one can fully utilize the information derived from the experimental results. In the case of genotyping arrays, for example, quantification of total DNA amount, relative allelic abundance, and prior information such as minor allele frequencies can be considered.

No apriori knowledge of the number of copy number states is required in the segmentation approach—an advantage in the study of cancer where polyploidy and contamination with normal tissues result in a wide range of fractional copy numbers. Possibly for the reasons outlined, HMMs are the methods of choice in the analysis of normal samples [10-14], while segmentation methods are the standard in cancer studies [15,16]. A limitation of segmentation methods is that they rely on data in which the variation in copy number is reflected in the differences in means of the segments. This fact makes segmentation methods applicable directly to a substantial portion of the data derived from recent technologies, but not to relative allelic abundance. However, see the modification suggested in [17] and the following description for an exception.

While a number of successful approaches have been derived along the lines described above, there is still a paucity of methodology for the joint analysis of multiple sequences. It is clear that if multiple subjects share the same variation in copy number, there exists the potential to increase power by joint analysis. Wang et al. [18] present a methodology that extends [1] to reconstruct the location of tumor suppressor genes from the identification of regions lost in a larger number of samples. The initial steps of the Birdsuite algorithm rely on the identification of suspect signals in the context of multiple sample. PennCNV [13] includes an option of joint analysis of trios. Methodology to process multiple samples within the context of change point analysis has been developed in [16,19-21]. Efron and Zhang [22] consider FDR analysis of independent samples to identify copy number polymorphisms (CNPs), and Nowak et al. [23] use a latent feature model to capture, in joint analysis of array-CGH data from multiple tumor samples, shared copy number profiles, on each of which a fused-lasso penalty is enforced for sparsity.

In the present work we consider a setting similar to [16] in that we want joint analysis to inform the segmentation of multiple samples. Our main focus is the analysis of genotyping array data, but the methodology we develop is applicable to a variety of platforms. By adopting a flexible framework we are able, for example, to define a segmentation algorithm that uses all information from Illumina genotyping data. As in [19], we are interested in the situation where some but not all the samples under consideration carry a copy number variant (CNV). We prefer to enforce a certain sparsity in the vector that identifies which samples carry a given variant. We tackle this problem using a penalized estimation approach, originally proposed in this context by [24], for which we have developed an algorithmic implementation [25]. Appreciable results are achieved in terms of speed, accuracy, and flexibility.

In concluding this introduction, we would like to make an important qualification. The focus of our contribution is on segmentation methods, knowing that this is only one of the steps necessary for an effective recovery of CNVs. In particular, normalization and transformation of the signal from experimental sources are crucial and can have a very substantial impact on final results, as documented in [26-31], for example. Indeed, preprocessing to eliminate systematic variation in intensities is particularly important for joint analysis of multiple sequences, when repeated deviances are more likely to be interpreted as true signal. Furthermore, calling procedures that classify results of segmentation while possibly controlling global error measures [22] are also needed. Indeed, in the data analysis included in this paper, we need to resort to both of these additional steps, and we will describe then briefly the fairly standard choices we make.

Before describing in detail the proposed methods for joint segmentation of multiple sequences, we start by illustrating various contexts where joint analysis appears to be useful.

### Genotyping arrays and CNV detection

Genotyping arrays have been used on hundreds of thousands of subjects. The data collected through them provides an extraordinary resource for CNV detection and the study of their frequencies in multiple populations. Typically, the raw intensity data (representing hybridization strength) is processed to obtain two signals: quantification of total DNA amount (from now on log R Ratio, LRR, following Illumina terminology) and relative abundance of the two queried alleles (from now on B allele frequency, BAF). Both these signals contain information on CNVs, and one of the strengths of HMMs has been that they can easily process them jointly. Segmentation models like CBS have traditionally relied only on LRR. While this is a reasonable choice, it can lead to substantial loss of information, particularly in tumor cells, where polyploidy and contamination make information in LRR hard to decipher. To exploit BAF in the context of a segmentation method, a signal transformation has been suggested [17]: mirrored BAF (mBAF) relies on exchangeability of the two alleles and the low information content of homozygous SNPs. The resulting mBAF is defined on a coarser grid than the original BAF, but is characterized by changing means in the presence of a CNV. While [17] shows that the analysis of BAF alone can be advantageous and more powerful than segmentation of LRR in some contexts, clearly a joint analysis of LRR and mBAF should be preferable to an arbitrary selection of one or the other signal.

### Multiple platforms

LRR and BAF are just one example of the multiple signals available in some samples. Often, as research progresses, the samples are assessed with a variety of technologies. For example, a number of subjects who have been genotyped at high resolution are now being resequenced. Whenever the technology adopted generates a signal that contains some information on copy number, there is an incentive to analyze the available signals jointly.

### Tumor samples from the same patient obtained at different sites or different progression stages

In an effort to identify mutations that are driving a specific tumor, as well as study its response to treatment, researchers might want to study CNVs in cells obtained at different tumor sites or at different time points [32]. Copy number is highly dynamic in cancer cells, so that it is to be expected that some differences will be detected over time or across sites. By contrast, the presence of the same CNVs across these samples can be taken as an indication that the tumors share the same origin. Therefore, a comparative analysis of CNV can be used to distinguish resurgence of the same cancer from insurgence of a new one, or to identify specific cancer cell populations. Given that the tissue extracted always consists of a mixture of normal and cancer cells, which are in turn a mixture of different populations, joint analysis of the signals from the varied materials is much more likely to lead to the identification of common CNVs when these exist.

### Related subjects

Family data is crucial in genetic investigations, and hence it is common to analyze related subjects. When studying individuals from the same pedigree, it is reasonable to assume that some CNVs might be segregating in multiple people and that joint analysis would reduce Mendelian errors and increase the power of detection.

The rest of the paper is organized as follows: In the Methods section, we first present the penalized estimation framework, and then describe how the model can be used for data analysis by: (a) outlining an efficient estimation algorithm, (b) generalizing it to the case of uncoordinated data, and (c) describing the choice of the penalization parameters. In the results section, we discuss our findings on two simulated data sets (descriptive of normal and tumor samples) and two real data sets. In one case multiple platforms are used to analyze the same sample, and in the other case samples from related individuals benefit from joint analysis.

## Methods

### A model for joint analysis of multiple signals

Assume we have observed *M* signals, each measured at *N* locations, corresponding to ordered physical positions along the genome, with *y*_*ij*_ being the observed value of sequence *i* at location *j*. The copy number process can be modeled as

(1) $$y_{\mathrm{ij}}=\beta_{\mathrm{ij}}+\varepsilon_{\mathrm{ij}},$$

where *ε*_*ij*_ represent noise, and the mean values *β*_*ij*_ are piece-wise constant. Thus, there exists a linearly ordered partition $\left\{R_{1}^{\left(i\right)},R_{2}^{\left(i\right)},\ldots ,R_{K_{i}}^{\left(i\right)}\right\}$ of the location index {1,2,…,*N*} such that $\beta_{\mathrm{is}}=\cdot \cdot \cdot =\beta_{\mathrm{it}}=\mu_{k}^{\left(i\right)}$ for $s,\ldots ,t\in R_{k}^{\left(i\right)}$ and 1 ≤ *k* ≤ *K*_*i*_. In other words, most of the increments |*β*_*ij*_ − *β*_*i*,*j*−1_ | are assumed to be zero. When two sequences *k* and *l* share a CNV with the same boundaries at location *j*, both |*β*_*kj*_ − *β*_*k*,*j*−1_ | and |*β*_*lj*_ − *β*_*l*,*j*−1_ | will be different from zero at the change point *j*. Modulo an appropriate signal normalization, *β*_*ij*_ = 0 can be interpreted as corresponding to the appropriate normal copy number equal to 2. We propose to reconstruct the mean values ***β***by minimizing the following function, called hereafter the generalized fused lasso (GFL):

(2) $$\begin{array}{lll} f(\beta ) & =\frac{1}{2}\overset{M}{\underset{i=1}{\sum }}\overset{N}{\underset{j=1}{\sum }}{\left(y_{\mathrm{ij}}-\beta_{\mathrm{ij}}\right)}^{2}+\lambda_{1}\overset{M}{\underset{i=1}{\sum }}\overset{N}{\underset{j=1}{\sum }}|\beta_{\mathrm{ij}}|\; & \;\\ & \;+\lambda_{2}\overset{M}{\underset{i=1}{\sum }}\overset{N}{\underset{j=2}{\sum }}|\beta_{\mathrm{ij}}-\beta_{i,j-1}|\; & \;\\ & \;+\lambda_{3}\overset{N}{\underset{j=2}{\sum }}{\left[\overset{M}{\underset{i=1}{\sum }}{\left(\beta_{\mathrm{ij}}-\beta_{i,j-1}\right)}^{2}\right]}^{\frac{1}{2}},\; \end{array}$$

which includes a goodness-of-fit term and three penalties, whose roles we will explain one at a time. The *ℓ*_1_ penalty $\overset{M}{\underset{i=1}{\sum }}\overset{N}{\underset{j=1}{\sum }}|\beta_{\mathrm{ij}}|$ enforces sparsity within ***β***, in favor of values *β*_*ij*_ = 0, corresponding to the normal copy number. The total variation penalty $\overset{N}{\underset{j=2}{\sum }}|\beta_{\mathrm{ij}}-\beta_{i,j-1}|$ minimizes the number of jumps in the piece-wise constant means of each sequence and was introduced by [24] in the context of CNV reconstruction from array-CGH data. Finally, the Euclidean penalty on the column vector of jumps $\sqrt{\overset{M}{\underset{i=1}{\sum }}{\left(\beta_{\mathrm{ij}}-\beta_{i,j-1}\right)}^{2}}$ is a form of the group penalty introduced by [33] and favors common jumps across sequences. As clearly explained in [34], “the local penalty around 0 for each member of a group relaxes as soon as the |*β*_*ij*_ − *β*_*i*,*j*−1_ | for one member *i* of the group moves off 0.” Bleakley and Vert (2011) [35] also suggested the use of this group-fused-lasso penalty to reconstruct CNV. We consider here the use of both the total variation and the Euclidean penalty on the jumps to achieve the equivalent effect of the sparse group lasso, which, as pointed out in [36], favors CNV detection in multiple samples, allowing for sparsity in the vector indicating which subjects are carriers of the variant. This property is important in situations of multiple tumor samples and related subjects, where one does not want to assume that all the *M* sequences carry the same CNV.

The incorporation of the latter two penalties can also be naturally interpreted in view of image denoising. To restore an image disturbed by random noise while preserving sharp edges of items in the image, a 2-D total variation penalty $\lambda \overset{M}{\underset{i=1}{\sum }}\overset{N}{\underset{j=2}{\sum }}|\beta_{\mathrm{ij}}-\beta_{i,j-1}|+\rho \overset{N}{\underset{j=1}{\sum }}\overset{M}{\underset{i=2}{\sum }}|\beta_{\mathrm{ij}}-\beta_{i-1,j}|$ is proposed in a regularized least-square model [37], where *β*_*ij*_ is the true underlying intensity of pixel (*ij*). In CNV detection problems, signals from multiple sequences can be aligned in the shape of an image, except that pixels in each sequence are linearly ordered while sequences as a group have no certain order a priori. Thus, one of the two total variation penalties is replaced by the group penalty on the column vector of jumps.

Using matrix notation, and allowing the tuning parameter *λ*_1_, *λ*_2_ and *λ*_3_ to be sequence specific, we can reformulate the objective function as follows. Let **Y** = (*y*_*ij*_)_*M*×*N*_ and ***β*** = (*β*_*ij*_)_*M*×*N*_. Let ***β***_*i*_ be the *i*th row of ***β*** and ***β***_(*j*)_ the *j*th column of ***β***. Also, let ***λ***_3_ = (*λ*_3,*i*_)_*M*×1_. Then we have

(3) $$\begin{array}{lll} f(\beta ) & =\frac{1}{2}||Y-\beta |{|}_{F}^{2}+\overset{M}{\underset{i=1}{\sum }}\lambda_{1,i}||\beta_{i}|{|}_{\ell_{1}}\; & \;\\ & \;+\overset{M}{\underset{i=1}{\sum }}\lambda_{2,i}||\beta_{i,2:N}-\beta_{i,1:(N-1)}|{|}_{\ell_{1}}\; & \;\\ & \;+\overset{N}{\underset{j=2}{\sum }}||\lambda_{3}*(\beta_{\left(j\right)}-\beta_{\left(j-1\right)})|{|}_{\ell_{2}},\; \end{array}$$

where ||·||_*F*_ is the Frobenius matrix norm, $||\cdot |{|}_{\ell_{1}}$ and $||\cdot |{|}_{\ell_{2}}$ are the *ℓ*_1_ and *ℓ*_2_ vector norms, ***β***_*i*,*s*:*t*_ indicates the sub-vector with elements *β*_*i*,*s*_,…,*β*_*i*,*t*_ of the row vector ***β***_*i*_, and “∗” denotes entry-wise multiplication between two vectors. It would be easy to modify the tuning parameters so as to make them location specific by reducing the penalty for a jump in genomic regions known to harbor CNVs.

### An MM algorithm

While the solution to the optimization problem (3) might have interesting properties, this approach is useful only if an effective algorithm is available. The last few years have witnessed substantial advances in computational methods for *ℓ*_1_ -regularization problems, including the use of coordinate descent [38,39] and path following methods [35,40-42]. The computational complexity of these methods in the best situation is *O*(*MNK*), where *k* indicated the number of knots along the solution path. Here knots are conjunction points between a series of piecewise functions of tuning parameters. It is important to note that these algorithms – some of which are designed for more general applications – may not be the most efficient for large scale CNV analysis for at least two reasons. On the one hand, reasonable choices of *λ* might be available, making it unnecessary to solve for the entire path; on the other hand, the number of knots *k* can be expected to be as large as *O*(*N*), making the computational costs of path algorithms prohibitive.

With specific regard to the fused-lasso application to CNV detection, we were successful in developing an algorithm with per iteration cost *O*(*N*) and empirically fast convergence rate for the analysis of one sequence [25]. We apply the same principles here and start by modifying the norms in the penalty to achieve better computational stability. For the *ℓ*_1_ norm we substitute $||x|{|}_{2,\varepsilon }=\sqrt{x^{2}+\varepsilon }$ for sufficiently small *ε*, and for the *ℓ*_2_ norm we substitute $||x|{|}_{2,\varepsilon }={\left(\overset{n}{\underset{i=1}{\sum }}x_{i}^{2}+\varepsilon \right)}^{\frac{1}{2}}$. This produces the differentiable objective function

(4) $$\begin{array}{lll} f_{\varepsilon }(\beta ) & =\frac{1}{2}\overset{M}{\underset{i=1}{\sum }}\overset{N}{\underset{j=1}{\sum }}{\left(y_{\mathrm{ij}}-\beta_{\mathrm{ij}}\right)}^{2}+\overset{M}{\underset{i=1}{\sum }}\lambda_{1,i}\overset{N}{\underset{j=1}{\sum }}||\beta_{\mathrm{ij}}|{|}_{2,\varepsilon }\; & \;\\ & \;+\overset{M}{\underset{i=1}{\sum }}\lambda_{2,i}\overset{N}{\underset{j=2}{\sum }}||\beta_{\mathrm{ij}}-\beta_{i,j-1}|{|}_{2,\varepsilon }\; & \;\\ & \;+\overset{N}{\underset{j=2}{\sum }}||\lambda_{3}*(\beta_{\left(j\right)}-\beta_{\left(j-1\right)})|{|}_{2,\varepsilon }.\; \end{array}$$

Adopting an MM framework [43], we want to find a surrogate function ${ℊ}_{\varepsilon }(\beta |\beta^{\left(m\right)})$ for each iteration *M* such that ${ℊ}_{\varepsilon }(\beta^{\left(m\right)}|\beta^{\left(m\right)})=f_{\varepsilon }(\beta^{\left(m\right)})$ and ${ℊ}_{\varepsilon }(\beta |\beta^{\left(m\right)})\geq f_{\varepsilon }(\beta )$ for all ***β***. At each iteration, the MM principle chooses $\beta^{\left(m+1\right)}=\text{arg min }{ℊ}_{\varepsilon }(\beta |\beta^{\left(m\right)})$. A majorizing function with the above properties is readily obtained using the concavity of the square-root function $\left||x|{|}_{2,\varepsilon }\leq \frac{1}{2||z|{|}_{2,\varepsilon }}(x^{2}-z^{2}\right)$, and its vector equivalent $\left||x|{|}_{2,\varepsilon }\leq \frac{1}{2||z|{|}_{2,\varepsilon }}(||x|{|}_{\ell_{2}}^{2}-||z|{|}_{\ell_{2}}^{2}\right)$. The resulting surrogate function

$$\begin{array}{lll} {ℊ}_{\varepsilon }(\beta |\beta^{\left(m\right)}) & =\frac{1}{2}\overset{M}{\underset{i=1}{\sum }}\overset{N}{\underset{j=1}{\sum }}{\left(y_{\mathrm{ij}}-\beta_{\mathrm{ij}}\right)}^{2}\; & \;\\ & \;+\overset{M}{\underset{i=1}{\sum }}\lambda_{1,i}\overset{N}{\underset{j=1}{\sum }}\frac{\beta_{\mathrm{ij}}^{2}}{2||\beta_{\mathrm{ij}}^{\left(m\right)}|{|}_{2,\varepsilon }}\; & \;\\ & \;+\overset{M}{\underset{i=1}{\sum }}\lambda_{2,i}\overset{N}{\underset{j=2}{\sum }}\frac{{\left(\beta_{\mathrm{ij}}-\beta_{i,j-1}\right)}^{2}}{2||\beta_{\mathrm{ij}}^{\left(m\right)}-\beta_{i,j-1}^{\left(m\right)}|{|}_{2,\varepsilon }}\; & \;\\ & \;+\overset{N}{\underset{j=1}{\sum }}\frac{||\lambda_{3}*(\beta_{\left(j\right)}-\beta_{\left(j-1\right)})|{|}_{\ell_{2}}^{2}}{2||\lambda_{3}*(\beta_{\left(j\right)}^{\left(m\right)}-\beta_{\left(j-1\right)}^{\left(m\right)})|{|}_{2,\varepsilon }}\;+\;c^{\left(m\right)}\; & \; \end{array}$$

separates as a sum of similar functions in the the row vectors ***β***_*i*_ ; namely,

$$\begin{array}{lll} {ℊ}_{\varepsilon }(\beta |\beta^{\left(m\right)}) & =\overset{M}{\underset{i=1}{\sum }}{ℊ}_{i}(\beta_{i}|\beta^{\left(m\right)}),\; & \; \end{array}$$

where

(5) $$\begin{array}{ll} {ℊ}_{i}(\beta_{i}|\beta^{\left(m\right)}) & =\frac{1}{2}\beta_{i}A_{i}^{\left(m\right)}\beta_{i}^{T}-{\left[b_{i}^{\left(m\right)}\right]}^{T}\beta_{i}^{T}+{\tilde{c}}_{i}^{\left(m\right)}.\; \end{array}$$

Here each $A_{i}^{\left(m\right)}$ is a tridiagonal symmetric matrix, and ${\tilde{c}}_{i}^{\left(m\right)}$ is a constant, irrelevant for optimization purposes. In view of the strict convexity of the surrogate function, each $A_{i}^{\left(m\right)}$ is also positive definite. The nonzero entries of $A_{i}^{\left(m\right)}$ and $b_{i}^{\left(m\right)}$ (*i* = 1,…,*M*) are listed in Additional file 1: Supplementary Text . Each of the surrogate functions in (5) can be minimized by solving the linear system $\beta_{i}={\left[\beta_{i}^{\left(m\right)}\right]}^{T}{\left[A_{i}^{\left(m\right)}\right]}^{-1}$ by the Tri-diagonal Matrix (TDM) algorithm [44]. This results in a per-iteration computational cost of *O*(*MN*). This algorithm is empirically observed to achieve an exponential convergence rate [25], although we do not yet have an analytic proof. In practice, this method scales well with joint analysis of tens to hundreds of samples with measurements at millions of locations, with limitations dictated by memory requirements. For analysis of real data, we suggest one or a group of samples to be analyzed chromosome by chromosome, since a CNV region can never extend beyond one chromosome to another. Actual computation times are shown along with different examples in the results section. Readers might be interested in comparing the outlined approach with other segmentation methods that are not based on the use of *ℓ*_1_ penalties, as [45].

### Stacking observations at different genomic locations

While copy number is continuously defined across the genome, experimental procedures record data at discrete positions, for which we have used the indexes *j*=1,…,*N*. In reality, repeated evaluations of the same sample (or related samples) will typically result in measurements at only partially overlapping genomic locations, either because different platforms use different sets of probes or missing data occur at different positions across the sequences. For example, for mBAF and LRR measurements from the same experiment on the same subject, the mBAF signal is defined on a subset of the locations where the LRR signal is defined.

Let *S* indicate the union of all genomic positions where some measurement is available among the *M* signals under study. And let *S*_*i*_ be the subset of locations with measurements in sequence *i*. We reconstruct *β*_*ij*_ for all *j* ∈ *S*. When *j* ∉ *S*_*i*_, *β*_*ij*_ will be determined simply on the basis of the neighboring data points, relying on the regularizations introduced in (3). The goodness-of-fit portion of the objective function is therefore redefined as

(6) $$\frac{1}{2}\overset{M}{\underset{i=1}{\sum }}\overset{N}{\underset{j=1}{\sum }}{\left(\delta_{\mathrm{ij}}y_{\mathrm{ij}}-\delta_{\mathrm{ij}}\beta_{\mathrm{ij}}\right)}^{2}\;\;\;\;\text{with}\;\;\;\;\delta_{\mathrm{ij}}=\left\{\begin{array}{ll} 1, & \text{if}\;\;j\in S_{i},\\ 0, & \text{otherwise}. \end{array}\right)$$

The MM strategy previously described applies with slight modifications of the matrix $A_{i}^{\left(m\right)}$ (see Additional file 1: Supplementary Text).

The attentive reader will have noted that the *y*_*ij*_ values with *j* ∉ *S*_*i*_ can be considered missing data, and evaluation of the missingness pattern is appropriate. In general, the *y*_*ij*_ cannot be considered missing at random. The most important example is the case of mBAF, where homozygous markers result in missing values. Homozygosity is clearly more common when copy number is equal to 1 than when copy number is equal to 2. Therefore, there is potentially more information on *β*_*ij*_ to be extracted from the signals than what we capture with the proposed method. Although most of the information on deletions is obtained through LRR, BAF does convey additional information on duplications, where the changes in LRR are limited by saturation effects. On the other hand, it does appear that our method does not increase the rate of false positives. Hence, it can be considered as an operational improvement over segmentation based on LRR only, even if in theory, it does not completely use the information on BAF.

### Choice of tuning constants and segmentation

One of the limitations of penalization procedures is that values for the tuning parameters need to be set, and clear guidelines are not always available. Path methods that obtain a solution of the optimization problem (3) for every value of a tuning parameter can be attractive, but recent algorithmic advances [35,41,42] remain impractical for problems of the size of ours. A number of recent publications obtain optimal values of penalty parameters under a series of conditions [46-49]. We rely on these findings to propose a strategy for obtaining a solution of (3) for reasonably liberal values of the tuning parameters, followed by a sequence-by-sequence hard thresholding of the detected jumps with a data-adaptive threshold.

We have found the following guidelines to be useful in choosing penalty parameter values:

(7) $$\begin{array}{lll} \lambda_{1,i} & =c_{1}{\hat{\sigma }}_{i},\; & \;\\ \lambda_{2,i} & =\rho (p)c_{2}{\hat{\sigma }}_{i}\sqrt{\text{log}\;N},\;\\ \lambda_{3,i} & =[1-\rho (p)]c_{3}{\hat{\sigma }}_{i}\sqrt{\mathrm{pM}}\sqrt{\text{log}\;N},\; & \; \end{array}$$

for *i* = 1,…,*M*, where ${\hat{\sigma }}_{i}$ is a robust estimate of standard deviation of **y**_*i*_, *p* is roughly the proportion of the *M* sequences we anticipate to carry CNVs, and *c*_1_, *c*_2_ and *c*_3_ are positive multipliers adjusted to account for different signal-to-noise ratios and CNV sizes. We discuss the function *ρ* below.

While a more rigorous justification is provided in the Additional file 1: Supplementary Text, we start by underscoring some of the characteristics of this proposal. 

● The sequence-specific penalizing parameters are proportional to an estimate of the standard deviation of the sequence signal. In other words, after initial normalization, the same penalties would be used across all signals.

● The tuning parameter for the total variation (fused lasso) and the Euclidean (group fused lasso) penalties on the jumps depend on $\sqrt{\text{log}\;N}$, where *N* is the possible number of jumps. This has a “multiple comparison controlling” effect and resembles rates that have been proven optimal under various sparse scenarios [46-49]. This term does not appear in the expression of *λ*_1_, as the lasso penalty can be understood as providing a soft thresholding of the solution of (3) when *λ*_1_ = 0. Given the penalization due to *λ*_2_ and *λ*_3_, the solution of (3) when *λ*_1_ = 0 will have much smaller dimension than *N*.

● The group penalty depends on $\sqrt{M}$, where *M* is the number of grouped sequences, as in the original proposal [33].

● The relative weight of the fused-lasso and group-fused-lasso penalties is regulated by *ρ*, which depends on *p*, the proportion of the *M* sequences expected to carry the same CNV. For example, if *M* = 2 and the two sequences record LRR and mBAF from the same individual, one expects *p* = 1 with *ρ* = 0, enforcing jumps at identical places in the two signals. Or, when studying the sequences relative to one parent and her/his offspring, it is reasonable to set *p* = 1/2, reflecting the fact that the two share half of their genome. For completely unrelated sequences, *p* = 0 and *ρ* = 1. In this setting, *p* is defined by genome-wide characteristic of the samples. However, when analyzing specific genomic segments, corresponding to copy number polymorphisms, it is possible to use *p* to reflect the population frequencies of each of the copy number variants. We do not consider the problem of estimating a variable *p* in the present work.

The standard deviation ${\hat{\sigma }}_{i}$ can be estimated robustly as follows. Let *Δ*_*ij*_ = *y*_*i*,*j* + 1_ − *y*_*i*,*j*_, for *j* = 1,…,*N* − 1, be the first-order differences of adjacent *y*_*ij*_ for sequence *i*. Then most $\text{Var}(\Delta_{\mathrm{ij}})=2\sigma_{i}^{2}$ except those bridging real change points, so we can take

$${\hat{\sigma }}_{i}=\hat{\mathrm{SD}}(\Delta_{i})/\sqrt{2},$$

where $\hat{\mathrm{SD}}(\Delta_{i})=\text{Standard Deviation}(\Delta_{i})$ or $\hat{\mathrm{SD}}(\Delta_{i})=\text{Median Absolute Deiviation}(\Delta_{i})$ for ***Δ***_*i*_ = {*Δ*_*i*,1_,…,*Δ*_*i*,*N*−1_ }.

As mentioned before, the exact values of the penalty parameters should be adjusted depending on the expectations of signal strengths. Following the approach in [50], one can approximate the bias induced by each of the penalties and hence work backwards in terms of acceptable levels. As detailed in Additional file 1: Supplementary Text,

$$\begin{array}{lll} \text{Bias}(\lambda_{1}) & \propto \lambda_{1}\; & \;\\ \text{Bias}(\lambda_{2}) & \propto \lambda_{2}/\text{Length of segment}\; & \;\\ \text{Bias}(\lambda_{3}) & \propto \lambda_{3}/\left(\text{Length of segment}\right)\; & \;\\ & \left(\;\times \sqrt{\text{\# sequences sharing segment}}\right)\; & \; \end{array}$$

Following again the approach in [50], one can show that under some relatively strong assumptions, the choices in (7) lead to a consistent behavior as N→∞ and *M* stays bounded (see Additional file 1: Supplementary Text). Despite the fact that *N* is indeed large in our studies, it is not clear that we can assume it to be in the asymptotic regime. As finer scale measurements become available, scientists desire to investigate CNVs of decreasing length. The CNVs we are interested in discovering are often covered by a small number of probes. Furthermore we have often little information on the sizes and frequencies of CNVs. In this context, we find it advisable to rely on a two-stage strategy: 

1. Sequences are jointly segmented by minimizing (3) for a relatively lax choice of the penalty parameters.

2. Jumps are further thresholded on the basis of a data-driven cut-off.

Step 2 allows us to be adaptive to the signal strength and can be carried on with multiple methods. For example, one can adopt the modified Bayesian Information Criteria (mBIC) [51]. For sequence *i*, the jumps are sorted as $\left\{{\hat{d}}_{i(1)},\ldots ,{\hat{d}}_{i(N-1)}\right\}$ in the descending order of their absolute values. Then we choose the first $\hat{k}$ change points where $\hat{k}$ is given by

$$\hat{k}=\underset{k}{\text{arg max }}\text{mBIC}\left(k\right).$$

In data analysis, we often apply an even simpler procedure where the threshold for jumps is defined as a fraction of the maximal jump size observed for every sequence. Specifically, for sequence *i*, let ${\hat{D}}_{i}={\text{max}}_{2\leq j\leq N}\{|{\hat{d}}_{\mathrm{ij}}|\}$, where ${\hat{d}}_{\mathrm{ij}}={\hat{\beta }}_{\mathrm{ij}}-{\hat{\beta }}_{i,j-1}$ is the largest observed jump for sequence *i*. Then we define

$$\gamma_{i}=\max\{a{\hat{\sigma }}_{i},\min\{{\hat{D}}_{i},b{\hat{\sigma }}_{i}\}\},\;\text{for}\;\;a<b,$$

as a “ruler” reflecting the scale of a possible real jump size, taking *cγ*_*i*_ as the cut-off in removal of most small jumps. In all analyses for this paper, we fix *a* = 1, *b* = 5 and *c* = 0.2. In our experience, this heuristic procedure works well for both tumor and normal tissue CNV data.

### Calling procedure

Even if this is not the focus of our proposal, in order to compare the performance of our segmentation algorithm with HMM approaches, it becomes necessary to distinguish gains from losses of copy number. While the same segmentation algorithm can be applied to a wide range of data sets, calling procedures depend more closely on the specific technology used to carry out the experiments. Since our data analysis relies on Illumina genotyping arrays, we limit ourselves to this platform and briefly describe the calling procedure adopted in the results section.

Analyzing one subject at the time, each segment with constant mean is assigned to one of five possible copy number states (*c* = 0,1,2,3,4). Let *R* collect the indexes of all SNPs comprising one segment and let (**x**_*R*_**y**_*R*_) = {(*x*_*j*_*y*_*j*_), *j* ∈ *R*} be the vectors of values for BAF and LRR in the segment. On the basis of typical patterns for BAF and LRR in the different copy number states (see [10,13,18]), we can write log-likelihood ratio

(8) $$\begin{array}{c} \mathrm{LR}(c)=\text{log}\;\frac{L_{\text{BAF}}(x_{R};c)}{L_{\text{BAF}}(x_{R};2)}+\text{log}\;\frac{L_{\text{LRR}}(y_{R};c)}{L_{\text{LRR}}(y_{R};2)},\;c=0,1,3,4, \end{array}$$

explicitly defined in Additional file 1: Supplementary Text. Segment *R* is assigned a CNV state ĉ that maximize LR(*c*), only if $\mathrm{LR}(ĉ)>r_{1}$, where *r*_1_ is a pre-specified cut-off.

As noted in [16], the LRR data for a segment with *c* = 2, ideally normalized to have mean 0, often has a small non-zero mean due to experimental artifacts. If the number of SNPs in *R* is sufficiently large, the above log-likelihood-ratio criterion would result in the erroneous identification of a copy number different from 2. To avoid this, we also require that the size of the absolute difference of the mean of LRR from zero be larger than a threshold $|{\bar{y}}_{R}|>r_{2}\sigma$.

## Results and discussion

We report the results of the analysis of two simulated and two real data sets, which overall exemplify the variety of situations where joint segmentation of multiple sequences is attractive, as described in the motivation section. In all cases, we compare the performance of the proposed procedure with a set of relevant, often specialized, algorithms. The penalized estimation method we suggest in this paper shows competitive performance in all cases and often a substantial computational advantage. Its versatility and speed make it a very convenient tool for initial exploration. To calibrate the run times reported in the sequel, we state for the record that all of our analyses were run on a Mac OS X (10.6.7) machine with 2.93 GHz Intel Core 2 Duo and 4 GB 1067 MHz DDR3 memory.

### Simulated CNV in normal samples

We consider one of the simulated datasets described in [25] with relatively short deletions and duplications (300 comprising 5, 10, 20, 30, 40, 50 SNPs each) are inserted in the middle of 13000 SNPs long sequences, using a combination of male and female X chromosome data from the Illumina HumanHap550 array, appropriately pre-processed to avoid biases. These steps included a scrambling of SNP positions so as to avoid long-range signal fluctuation. This setting mimics the small rare CNVs possibly occurring in the genome of normal individuals. In our main analysis, therefore, we process one individual at the time, reflecting the typical level of information available to scientists in these contexts. HMM methods, like PennCNV, are expected to be the most effective in this problem; segmentation methods like CBS are closer to our own and therefore also make an interesting comparison. As repeatedly discussed, the Illumina platform produces two signals for one subject, LRR and BAF. A segmentation method that can process one signal at the time would give its best results using LRR, which carries most of the information. Given this background, we compare four methods: PennCNV, CBS on LRR, fused lasso on LRR only, and group fused lasso on LRR and mBAF. The implementations we use are those reflected in the software packages: PennCNV (version 2010May01), R package DNAcopy for CBS (version 1.24.0) [52] and our own R package Piet (version 0.1.0). Tuning parameters for PennCNV and CBS are set at the default values; the fused lasso implementation corresponds to *λ*_1_ = 0.1, $\lambda_{2}=2\times \sqrt{13000}$, and *λ*_3_ = 0 and the group fused lasso to *λ*_1_ = 0.1, *λ*_2_ = 0, and $\lambda_{3}=2\times \sqrt{13000}$. To call deletions and duplications with CBS and the two fused-lasso approaches, we use both LRR and BAF data (prior to transformation to mBAF) with the following cut-off values: *r*_1_ = 10 and *r*_2_ = 1(1.5) for duplication (deletion). Performance is evaluated by the same indexes we used in [25], the true positive rate (TPR or sensitivity) and the false discovery rate (FDR), all defined on a per SNP basis. Results are summarized in Table 1.

**Table 1:** Detection accuracy and computation time of four methods on simulated normal samples

**CNV size**	**CNV type**		**PennCNV**			**CBS**			**Fused Lasso**			**Group Fused Lasso**	
			**TPR**	**FDR**		**TPR**	**FDR**		**TPR**	**FDR**		**TPR**	**FDR**
5	Deletion		83.80	4.92		78.20	0.68		63.93	1.74		64.27	1.83
	Duplication		58.53	4.67		11.67	10.26		20.00	37.76		39.87	14.33
10	Deletion		95.03	1.45		88.37	0.56		88.50	0.60		88.87	0.56
	Duplication		93.43	0.78		56.50	4.40		83.90	12.60		91.60	3.85
20	Deletion		94.63	0.58		90.50	0.39		90.80	0.47		90.83	0.47
	Duplication		96.13	0.92		86.22	3.58		92.77	4.95		94.98	2.13
30	Deletion		94.57	0.28		93.30	0.29		89.38	0.52		89.77	0.53
	Duplication		96.09	0.05		90.77	1.61		94.32	1.78		94.98	1.29
40	Deletion		97.83	0.59		97.58	0.09		97.28	0.19		97.28	0.19
	Duplication		94.61	0.46		92.77	0.98		93.94	1.15		94.63	0.75
50	Deletion		94.33	0.07		92.76	0.04		90.47	0.11		90.48	0.11
	Duplication		94.50	0.09		93.81	0.74		93.11	0.79		93.64	0.49
Overall Deletion			95.02	0.55		93.06	0.19		91.08	0.33		91.19	0.34
Overall Duplication			93.82	0.44		86.92	1.55		90.56	2.85		92.46	1.38
Overall			94.42	0.49		89.99	0.85		90.82	1.60		91.83	0.87
Time (sec.)			0.48 (0.01)			0.78 (0.69)			0.22 (0.13)			0.28 (0.05)	

TPR and FDR are measured as the percentage of related SNPs. Overall accuracy is calculated by pooling all sequences with a given type of CNV. Also reported are the average and standard deviation of the number of seconds required for the analysis of one sequence.

Not surprisingly, all algorithms perform similarly well for larger deletions/duplications, and it is mainly for variants that involve 10 or fewer SNPs that differences are visible. Algorithms that rely only on LRR (for example, CBS and fused lasso) underperform in the detection of small duplications. Comparison is particularly easy for duplications involving 10 SNPs, where the selected parameter values lead to similar FDRs in the three segmentation methods. The group fused lasso can almost entirely recover the performance of PennCNV and outperforms CBS in this context.

Out of curiosity, we analyzed all sequences simultaneously. While this represents an unrealistic amount of prior information, it allows us to evaluate the possible advantages of joint analysis. FDR practically became 0 (<0.02%) for all CNV sizes, but power increases only for CNVs including fewer than 10 SNPs.

Finally, it is useful to compare running times. Summary statistics of the per sample time are reported in Table 1. While all algorithms are rather fast, the two implementations of the fused lasso dominate.

### A simulated tumor data set

To explore the challenges presented by tumor data, we rely on a data set created by [17], with the specific goal of studying the effect of contamination between normal and cancer cells. The HapMap sample NA06991, genotyped on the Illumina HumanHap550 array, was used to simulate a cancer cell line by inserting a total of 10 structure variation regions, including one-copy losses, one-copy gains, and copy neutral loss-of-hetrozygosity (CN-LOH) (see Additional file 2: Table S1). The signal from this artificial “tumor” sample was then contaminated in silico with that of the original “normal” sample, resulting in 21 data sets, with a percentage of normal cells ranging from 0% to 100%. Note that most simulated CNV or CN-LOH regions are very large (some spanning an entire chromosome), and the challenge in detection is really due to the contamination levels.

For ease of comparison, we evaluate the accuracy of calling procedures as in the original reference [17]. Sensitivity is measured for each variant region as the percentage of heterozygous SNPs that are assigned the correct copy number, and specificity is measured as the percentage of originally heterozygous SNPs in unperturbed regions that are assigned CN=2. We compare the performance of GFL to BAFsegmentation [17] and PSCN [53] representing, respectively, a version of segmentation and HMM approaches specifically developed to deal with contaminated tumor samples. Both of these algorithms have been tested with success on this simulated data set.

Following other analyses, we do not pre-process the data prior to CNV detection. BAFsegmentation and PSCN were run using recommended parameter values. For each of the diluted data sets, we applied the GFL model on each chromosome, simultaneously using both LRR and mBAF, whose standard deviations are normalized to 1. Tuning constants are set to *λ*_1_ = 0, $\lambda_{2}=0.5\times 3\times \sqrt{\text{log}\;N}$, and $\lambda_{3}=0.5\times 3\times \sqrt{\text{log}\;N}$ for chromosomes interrogated by *N* SNPs. The change points resulting from hard segmentation on LRR and mBAF are combined to make a finer segmentation of the genome. Finally, we adopt the same calling procedure described by [17]. For ease of comparison with PSCN, only analyses of simulated tumor data are reported, even if BAFsegmentation and GFL would gain from using the genotype of the normal cell in defining mBAF.

**Figure 1:**
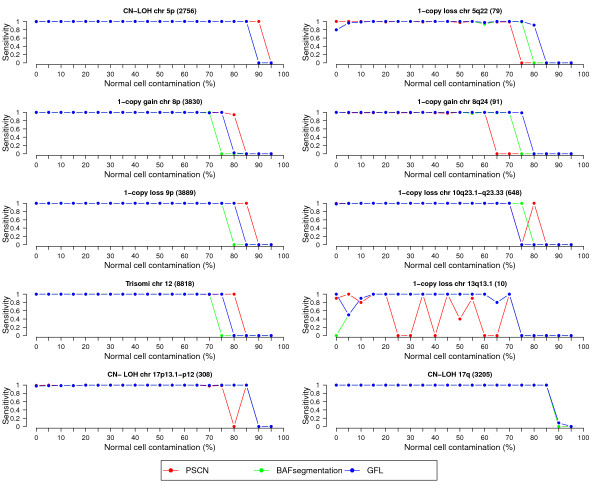
**Sensitivity as a function of percentage contamination by normal cells in the 10 different simulated CNV regions.** Sensitivity is not defined at 100% contamination.

**Figure 2:**
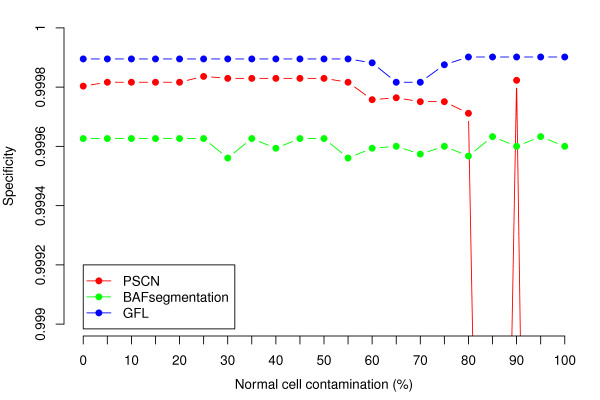
**Specificity as a function of percentage contamination by normal cells.** Note that [53] reports better performance of PSCN in dealing with contamination levels of 85%, 95% and 100%.

Figure 1 summarizes the sensitivity of each method, as a function of the percentage of normal cells in the sample. Sensitivity is calculated for each of the 10 regions separately. All three methods work reasonably well under a wide range of percentages of normal cell contamination. In 5 out of the 10 regions, GFL appears to lead to best results, while in the other 5, PSCN does. The CNV region involving the fewest SNPs is the hemizygous loss on Chromosome 13. In this case GFL in our hands behaved in the most stable manner. GFL outperforms the two comparison methods in terms of specificity (Figure 2). While the specificity values might appear very high in any case, this is somewhat an artifact of how we define this index. In the interest of fairness, it is relevant to note that the performance of PSCN in our hands did conform to the published performance [53]. While we tried our best to set the parameter values, we have not succeeded in replicating the authors’ original results.

PSCN, like GFL, is implemented in R with some computationally intensive subroutines coded in C. BAFsegmentation relies on the R package DNAcopy, whose core algorithms are implemented in C and Fortran. BAFsegmentation wraps these in Perl. A comparison of run times indicate that GLF and BAFsegmentation are comparable, while PSCN is fifty times slower than GFL (see Additional file 3: Table S2).

### One sample assayed with multiple replicates and multiple platforms

We use the data from a study [54] assessing the performance of different array platforms and CNV calling methods to illustrate the advantages of joint analysis of multiple measurements on the same subject. DNA from four individuals was analyzed in triplicate on each of 5 platforms: Affymetrix 6.0, Illumina 1M, 660W, Omni1-Quad (O1Q) and Omni2.5-Quad (O2Q) (among others [54]). We use the results on the first three to define “true” copy numbers and try to reconstruct them using data from O1Q and O2Q. The nine “reference” experiments were analyzed with 4 or 5 CNV calling algorithms [54] and a CNV was identified using majority votes. Consistent evidence was required from at least 2 analysis tools, on at least 2 platforms, and in at least 2 replicates (see Additional file 4: Table S3). Here overlapping CNVs detected in two replicates/algorithms/platforms are collapsed to a single CNV.

The test experiments are based on 1,020,596 and 2,390,395 autosomal SNPs, which after quality control reduce to a total of 2,657,077 unique loci. Since our focus here is to investigate how to best analyze multiple signals on the same subject, rather than on the specific properties of any CNV calling method, we carry out all the analyses using different settings of GFL in segmentation while keeping the same CNV calling and summarizing procedures. All segmentation is done on LRR only, while calling procedure uses both LRR and BAF (with cut-off *r*_1_ = 10 and *r*_2_ = 1). Here we compare three segmentation settings to analyze these 6 experiments per subject (see Additional file 5: Table S4 for more details about tuning parameters): 

1. The signals from the three technical replicates with one platform are averaged and then segmented and subjected to calling procedure separately. The final CNV list is the union of CNV calls from the two platforms.

2. The signals from the three technical replicates with one platform are each segmented and separately subjected to calling. A majority vote of at least two out of three is used to summarize each CNV result for each platform. The final CNV list is the union of the two platforms’ lists.

3. The signals from the three technical replicates of both platforms (6 LRR sequences) are segmented jointly. Calling is still done on each replicate separately, and the same majority vote is used to summarize the CNV result for each platform. Again, the final CNV list is the union of the two platforms’ results.

To benchmark the result of joint analysis, we use MPCBS [20], a segmentation method, specifically designed for multi-platform CNV analysis. The segments output from MPCBS are subjected to the same calling, majority voting, and summarizing procedures.

Table 2 presents our results. Averaging from different technical replicates leads to loss of power, while joint analysis of all the signals leads to the most effective performance. GFL joint analysis leads to results comparable to those of MPCBS, but it is at least 30 times faster than MPCBS.

**Table 2:** Comparison of four CNV analyses on four real normal samples

**Analysis**		**NA15510**			**NA18517**			**NA18576**			**NA18980**			**Time (min.)**
		**#Det.**	**#Ovlp.**		**#Det.**	**#Ovlp.**		**#Det.**	**#Ovlp**		**#Det.**	**#Ovlp**		
Analysis 1		170	38		144	34		160	25		145	22		1.2
Analysis 2		102	36		109	33		93	25		91	20		3.7
Analysis 3		80	38		82	32		69	25		56	15		8.5
MPCBS		98	34		88	28		59	18		68	21		313.9

The number of CNV detected (Det.) and overlapping (Ovlp.) and the average computation time (in minutes) for each sample under the different analyses.

### Multiple related samples assayed with the same platform

In the context of a study of the genetic basis of bipolar disorder, the Illumina Omni2.5-Quad chip was used to genotype 455 individuals from 11 Columbian and 13 Costa Rican pedigrees. We use this data set to explore the advantages of a joint segmentation of related individuals. In the absence of a reference evaluation of CNV status in these samples, we rely on two indirect methods to assess the quality of the predicted CNVs. We used the collection of CNVs observed in HapMap Phase III [5] to compile a list of 426 copy number polymorphisms (selecting all those CNVs with frequency ≥0.05 in pooled samples from 11 populations) and assumed that if we identify in our sample a CNV corresponding to one of these regions, we should consider it a true positive. For the purposes of this analysis we considered a detected CNV to correspond to one identified in HapMap if there was any overlap between the two regions.

Another indirect measure of the quality of CNV calls derives from the number of Mendelian errors encountered in the pedigrees when we consider the CNV as a segregating site. De novo CNVs are certainly a possibility, and in their case Mendelian errors are to be expected. However, when the CNV in question is a common one (already identified in HapMap), it is reasonable to expect that it segregates in the pedigrees as any regular polymorphism. We selected a very common deletion on Chromosome 8 (HapMap reports overall frequency >0.4 in 11 populations) and compared different CNV calling procedures on the basis of how many Mendelian errors they generate.

As mentioned before, PennCNV represents a state-of-the-art HMM method for the analysis of normal samples and, therefore, we included it in our comparisons. However, the parameters of the underlying HMM algorithm had not been tuned on the Omni2.5-Quad at this time, resulting in sub-standard performance. Segmentation methods are less dependent on parameter optimization; hence, GFL analysis of LRR and BAF one subject at a time can provide a better indication of the potential of single-sample methods. We considered two multiple-sample algorithms: GFL and MSSCAN [16], both applied on LRR with the group structure defined by pedigree membership. (While a trio-mode is available in PennCNV [55], this does not adapt to the structure of our families.) A final qualification is in order. While the authors of MSSCAN kindly shared with us a beta-version of their software, we did not find it to be robust. Indeed, we were unable to use it to segment the entire genome. However, we successfully used it to segment Chromosome 8, so that we could include MSSCAN in the comparison based on Mendelian errors.

Prior to analysis, the data was normalized using the GC-content correction implemented in PennCNV [29]. For individual analysis, the GFL parameters were *λ*_1_ = 0.1, *λ*_2_ = 0, and $\lambda_{3}=2\times \sqrt{\text{log}\;N}$, where *N* is the number of SNPs deployed on each chromosome; for pedigree analysis, the GFL parameters were *λ*_1_ = 0.1, $\lambda_{2}=0.5\times 2\times \sqrt{\text{log}\;N}$, and $\lambda_{3}=0.5\times 2\times \sqrt{0.3M}\times \sqrt{\text{log}\;N}$, where *M* is the number of individuals in each pedigree. For MSSCAN, CNV size is constrained to be fewer than 200 SNPs, and the maximum number of change points is set at 50. The calling procedure with *r*_1_ = 10 and *r*_2_ = 1 was applied to both the GFL and MSSCAN results.

Table 3 summarizes the total number of copy number polymorphisms (CNPs) identified in our sample by different approaches and their overlap with known CNPs from HapMap. For the purpose of this comparison we considered a variant to be a CNP when its frequency in our sample was at least 10%. All analysis modes of GFL show a higher percent of overlap with the HapMap list than the PennCNV list. It is also clear that GFL pedigree analysis achieves a larger overlap with the HapMap data than the GFL individual analysis. The time cost per sample is reasonable and scales well with the increment of sample size.

**Table 3:** Comparison of three CNV analyses in the bipolar disorder study

**Method**	**#Detected CNVR**	**#Overlap**	**%Overlap**	**Time (min.)**
PennCNV	189	63	33.33%	3.44
GFL-Individual (LRR+BAF)	95	50	52.63%	3.90
GFL-Pedigree (LRR)	106	62	58.49%	1.57

The number and overlap of CNP regions with frequency ≥0.1 detected in our sample by different methods. These CNP regions were compiled from HapMap. Computation time is given in minutes per sample.

**Figure 3:**
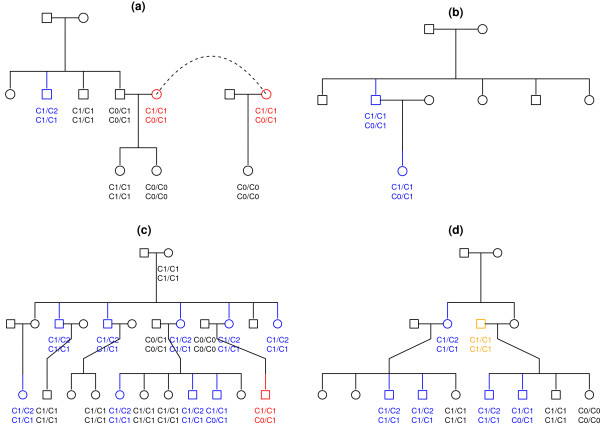
**CNV detection and Mendelian errors for a Central American pedigree.** Displayed are four families derived from an extended pedigree. Circles and squares correspond to females and males. The dashed line is used to indicate identical individuals. Beneath each individual, from top to bottom, are CNV genotypes by PennCNV and by GFL. The subjects for whom PennCNV and GLF infer different CNV genotypes are highlighted in red and blue. Red is used when PennCNV genotypes result in Mendelian error, while GFL genotypes do not. Blue is used when both genotypes are compatible with Mendelian transmissions. Orange singles out a member for whom both PennCNV and GFL genotypes result in Mendelian error.

Table 4 summarizes the results of our investigation of a 154kb CNP region on Chromosome 8p (from 39,351,896 to 39,506,122 on NCBI Build 36 coordinate). All methods but PennCNV show detected deletions only; this coincides with the observation from HapMap data. We used option *Mistyping* of Mendel (version 11.0) [56,57] to detect Mendelian errors. Joint segmentation methods discover more hemizygous deletions than individual analysis, resulting in fewer Mendelian errors. MSSCAN discovers the largest number of hemizygous deletions. Figure 3 shows four families derived from a large pedigree where 3 out of 4 Mendelian errors are removed by joint analysis.

**Table 4:** Detected CNVs in a common deletion on Chromosome 8

**Method**	**#CN= 0**	**#CN=1**	**#CN=3**	**#Families with Mendelian errors**	**Time (min.)**
PennCNV	125	39	102	35	0.19
GFL-Individual	123	97	0	20	0.21
GFL-Pedigree	123	137	0	15	0.09
MSSCAN-Pedigree	123	154	0	15	0.11

Across the various algorithms, subjects are assigned to one of 4 copy numbers. For each algorithm, we report the total numbers of CN≠2 identified, the total number of nuclear families with Mendelian errors, and the average computation time (in minutes) per sample for the analysis of Chromosome 8.

## Conclusions

We have presented a segmentation method based on penalized estimation that is capable of processing multiple signals jointly. We have shown how this leads to improvements in the analysis of normal samples (where segmentation can be applied to both total intensity and allelic proportions), tumor samples (where we are able to deal with contamination effectively), measurements from multiple platforms, and related individuals. Given that copy number detection is such an active area of research, it is impossible to compare one method to all other available methods. However, for each of the situations we analyzed, we tried to select state of the art alternative approaches. In comparison to these, the algorithm we present performs well. Its accuracy is always comparable to that of the most effective competitor and its computation times are better contained. Given its versatility and speed, GFL is, in our opinion, particularly useful for initial screening.

There are of course many aspects of CNV detection, ranging from normalization and signal transformation to FDR control of detected CNV, that we have not analyzed in this paper. There are also a number of improvements to our approach that appear promising, but at this stage are left for further work. For example, it is easy to modify algorithms so that the penalization parameters are location dependent and incorporate prior information on known copy number polymorphisms. It will be more challenging to develop theory and methods to select the values of these regularization parameters in a data-adaptive fashion.

Finally, while our scientific motivation has been the study of copy number variations, the joint segmentation algorithm we present is not restricted to specific characteristics of these data types, and we expect it will be applied in other contexts.

### Implementation and availablity

We have implemented the segmentation routine, which is our core contribution, in an R package (Piet) available at R-forge [58]. To demonstrate a visualization of the CNV results on Chromosome 8 in the bipolar disorder study, we refer readers to Additional file 6: Figure S1.

## Abbreviations

BAF: B allele frequency; CN: Copy number; CNV: Copy number variant; CNP: Copy number polymorphism; CN-LOH: Copy neutral loss of heterozygosity; GFL: Generalized fused lasso; HMM: Hidden Markov model; LRR: Log R ratio; MML: Majorization-minimization.

## Competing interests

The authors declare that they have no competing interests.

## Author’s contributions

ZZ, KL, and CS conceived this study and participated in model and algorithm development. ZZ performed the statistical analysis and wrote the R Piet implementation. All authors participated in writing the final manuscript. All authors read and approved the final manuscript.

## Supplementary Material

Additional file 1 **Supplementary Text.** Specification of surrogate function, justification of choice of tuning parameters, details of calling procedure. Click here for file

Additional file 2 **Table S1.** Regions of allelic imbalance imputed to the HapMap sample NA06991. Click here for file

Additional file 3 **Table S2.** Speed comparison of three methods: GFL, BAFsegmentation and PSCN. Click here for file

Additional file 4 **Table S3.** Sample information and reference CNV regions summarized for each sample by their types and sizes. Click here for file

Additional file 5 **Table S4.** Summary of results for four real samples under different CNV analyses. Click here for file

Additional file 6 **Figure S1.** Visualization of pedigree-wise CNV analysis results of Chromosome 8 data in the bipolar disorder study. In the main body of the plot, CNVs estimated for each individual are marked by small segments with color code: CN= 0 in blue, CN=1 in light blue, CN=3 in red and CN=4 in brown. Each subject is a row, each SNP a column. Subjects belonging to the same pedigree are stacked together. The pedigree names are indicated on the left-hand side with the number of pedigree members included in parentheses. On the right-hand side, the barplot represents the number of CNVs detected per subject. Two shades of green are switched alternately to indicate the pedigree to which the subject belongs. At the bottom, the gray histogram shows the GC content along the chromosome. Coordinated with the representation of CNVs in the main body, the green histogram counts the frequency of CNVs among the subjects represented. Vertical dotted line marks the centromere. Click here for file
