# Supplementary material for: Reconstructing DNA copy number by joint segmentation of multiple sequences
Source: BMC Bioinformatics. 2012 Aug 16;13:205. doi: 10.1186/1471-2105-13-205 (PMC3534631; doi:10.1186/1471-2105-13-205)
Supplement: Additional file 1 — Supplementary Text. Specification of surrogate function, justification of choice of tuning parameters, details of calling procedure. [file 1471-2105-13-205-S1.pdf]

Reconstructing DNA Copy Number by Joint Segmentation of Multiple Sequences

SUPPLEMENTARY TEXT

Zhongyang Zhang, Kenneth Lange, and Chiara Sabatti

March 2012

TDM algorithm

The non-zero entries in $\mathbf{A}_i$ and $\mathbf{b}_i$ in the re-shaped surrogate function (5) in the main text are listed as follows:

$$
\begin{aligned}
a_i^{(m)}(1,1) &= 1 + \frac{\lambda_{1,i}}{||\beta_{i1}^{(m)}||_{2,\epsilon}} + \frac{\lambda_{2,i}}{||\beta_{i2}^{(m)} - \beta_{i1}^{(m)}||_{2,\epsilon}} + \frac{\lambda_{3,i}^2}{||\boldsymbol{\lambda}_3 * (\boldsymbol{\beta}_{(2)}^{(m)} - \boldsymbol{\beta}_{(1)}^{(m)})||_{2,\epsilon}}; \\
a_i^{(m)}(j,j) &= 1 + \frac{\lambda_{1,i}}{||\beta_{ij}^{(m)}||_{2,\epsilon}} + \frac{\lambda_{2,i}}{||\beta_{ij}^{(m)} - \beta_{i,j-1}^{(m)}||_{2,\epsilon}} + \frac{\lambda_{2,i}}{||\beta_{i,j+1}^{(m)} - \beta_{ij}^{(m)}||_{2,\epsilon}} \\
&\quad + \frac{\lambda_{3,i}^2}{||\boldsymbol{\lambda}_3 * (\boldsymbol{\beta}_{(j)}^{(m)} - \boldsymbol{\beta}_{(j-1)}^{(m)})||_{2,\epsilon}} + \frac{\lambda_{3,i}^2}{||\boldsymbol{\lambda}_3 * (\boldsymbol{\beta}_{(j+1)}^{(m)} - \boldsymbol{\beta}_{(j)}^{(m)})||_{2,\epsilon}}, \\
&\qquad j = 2, \ldots, n-1; \\
a_i^{(m)}(n,n) &= 1 + \frac{\lambda_{1,i}}{||\beta_{in}^{(m)}||_{2,\epsilon}} + \frac{\lambda_{2,i}}{||\beta_{in}^{(m)} - \beta_{i,n-1}^{(m)}||_{2,\epsilon}} + \frac{\lambda_{3,i}^2}{||\boldsymbol{\lambda}_3 * (\boldsymbol{\beta}_{(n)}^{(m)} - \boldsymbol{\beta}_{(n-1)}^{(m)})||_{2,\epsilon}}; \\
a_i^{(m)}(j,j-1) &= -\frac{\lambda_{2,i}}{||\beta_{ij}^{(m)} - \beta_{i,j-1}^{(m)}||_{2,\epsilon}} - \frac{\lambda_{3,i}^2}{||\boldsymbol{\lambda}_3 * (\boldsymbol{\beta}_{(j)}^{(m)} - \boldsymbol{\beta}_{(j-1)}^{(m)})||_{2,\epsilon}}, \quad j = 2, \ldots, n; \\
a_i^{(m)}(j,j+1) &= -\frac{\lambda_{2,i}}{||\beta_{i,j+1}^{(m)} - \beta_{ij}^{(m)}||_{2,\epsilon}} - \frac{\lambda_{3,i}^2}{||\boldsymbol{\lambda}_3 * (\boldsymbol{\beta}_{(j+1)}^{(m)} - \boldsymbol{\beta}_{(j)}^{(m)})||_{2,\epsilon}}, \quad j = 1, \ldots, n-1; \\
b_i^{(m)}(j) &= y_{ij}, \quad j = 1, \ldots, n.
\end{aligned}
$$

When staking measurements at different positions, the item 1 in $a_i^{(m)}(j,j)$ is replaced by δ_{ij} and $b_i^{(m)} = y_{ij}$ is replaced by $b_i^{(m)} = \delta_{ij} y_{ij}$.

Bias estimation

Let x_{ij} be the data for sequence i at locus j after σ_i of each sequence is normalized to 1. With such normalization, the model (3) in the main text is reduced to a simpler form with global tuning parameters to each sequence for easier interpretation:

$$
f(\boldsymbol{\beta}) = \frac{1}{2}\sum_{i=1}^{M}\sum_{j=1}^{N}(x_{ij}-\beta_{ij})^2 + \lambda_1 \sum_{i=1}^{M}\sum_{j=1}^{N}|\beta_{ij}| + \lambda_2 \sum_{i=1}^{M}\sum_{j=2}^{N}|\beta_{ij}-\beta_{i,j-1}| + \lambda_3 \sum_{j=2}^{N}\left[\sum_{i=1}^{M}(\beta_{ij}-\beta_{i,j-1})^2\right]^{\frac{1}{2}}. \tag{S.1}
$$

The solution to minimize $f(\boldsymbol{\beta})$ is unique for $f(\boldsymbol{\beta})$ is strictly convex. Denote the solution as $\hat{\boldsymbol{\beta}} = (\hat{\beta}_{ij})_{M\times N}$. Suppose sequence i is partitioned into $\hat{K}_i$ consecutive segments $\{\hat{R}_1^{(i)}, \ldots, \hat{R}_{\hat{K}_i}^{(i)}\}$, delimited with change points $\hat{\mathcal{J}}_i = \{\hat{j}_1^{(i)}, \ldots, \hat{j}_{\hat{K}_i-1}^{(i)}\} \subset \{2, \ldots, N\}$ (left end of segment $2, \ldots, \hat{K}_i$). The fitted means of each segment is denoted as $\hat{\boldsymbol{\mu}}^{(i)} = (\hat{\mu}_1^{(i)}, \ldots, \hat{\mu}_{\hat{K}_i}^{(i)})$, i.e., $\hat{\beta}_{ij} = \hat{\mu}_k^{(i)}$, if $j \in \hat{R}_k^{(i)}$. The length (number of SNPs) of each segment is $\hat{L}_k^{(i)} = |\hat{R}_k^{(i)}|, k = 1, \ldots, \hat{K}_i$. Thus, the estimated mean vector for sequence i can be written as

$$\hat{\boldsymbol{\beta}}_i = \sum_{k=1}^{\hat{K}_i} \hat{\mu}_k^{(i)} I_{\hat{R}_k^{(i)}}.$$

$\hat{\boldsymbol{\beta}}$ is the optimal solution if and only if it satisfies the subgradient condition $\partial f(\hat{\boldsymbol{\beta}}) = 0$, i,e.,

$$\hat{\beta}_{ij} = y_{ij} - \lambda_1 s_{ij}^{(1)} - \lambda_2 s_{ij}^{(2)} - \lambda_3 s_{ij}^{(3)}, \tag{S.2}$$

where $s_{ij}^{(1)}$, $s_{ij}^{(2)}$ and $s_{ij}^{(3)}$ are coordinates of subgradient corresponding to β_{ij}'s appearing in each of the three penalty terms. Both bias estimation and asymptotic analysis rely on the analytic form of subgradient. Now we discussed the bias induced by each penalty separately.

Bias induced by lasso penalty

It is easy to verify that the subgradient for the lasso penalty can be written as

$$s_{ij}^{(1)} = \text{sign}(\beta_{ij}),$$

where, with a bit abuse of notation,

$$\text{sign}(x) = \begin{cases} 1, & \text{if } x > 0, \\ -1, & \text{if } x < 0, \\ z \in [-1, 1], & \text{if } x = 0. \end{cases} \tag{S.3}$$

Hence, the lasso penalty term merely plays as a soft-thresholding on the fitted values resulted from the model (S.1) with $\lambda_1 = 0$, denoted as $\hat{\beta}_{ij}(0, \lambda_2, \lambda_3)$, that is, for any $\lambda_1 > 0$,

$$\hat{\beta}_{ij}(\lambda_1, \lambda_2, \lambda_3) = \text{sign}\left[\hat{\beta}_{ij}(0, \lambda_2, \lambda_3)\right]\left[\hat{\beta}_{ij}(0, \lambda_2, \lambda_3) - \lambda_1\right]_+,$$

where $(x)_+ = \max\{x, 0\}$. This is also highlighted in Lamma A.1 of [2] for model (S.1) with $\lambda_3 = 0$.

Bias induced by fused-lasso penalty

In model (S.1) with $\lambda_1 = 0$ and $\lambda_3 = 0$, i.e., only fused-lasso penalty involved, Lemma 2.1 in [3] gives a insightful characterization of $\hat{\boldsymbol{\mu}}^{(i)}$:

$$\hat{\mu}_k^{(i)} = \frac{1}{\hat{L}_k^{(i)}} \sum_{j \in \hat{R}_k^{(i)}} x_{ij} + \hat{c}_k^{(i)}, \quad k = 1, \ldots, \hat{K}_i,$$

where

$$\hat{c}_1^{(i)} = \begin{cases} -\frac{\lambda_2}{\hat{L}_1^{(i)}}, & \text{if } \hat{\mu}_2^{(i)} - \hat{\mu}_1^{(i)} > 0, \\ \frac{\lambda_2}{\hat{L}_1^{(i)}}, & \text{if } \hat{\mu}_2^{(i)} - \hat{\mu}_1^{(i)} < 0, \end{cases}$$

$$\hat{c}_{\hat{K}_i}^{(i)} = \begin{cases} \frac{\lambda_2}{\hat{L}_{\hat{K}_i}^{(i)}}, & \text{if } \hat{\mu}_{\hat{K}_i}^{(i)} - \hat{\mu}_{\hat{K}_i-1}^{(i)} > 0, \\ -\frac{\lambda_2}{\hat{L}_{\hat{K}_i}^{(i)}}, & \text{if } \hat{\mu}_{\hat{K}_i}^{(i)} - \hat{\mu}_{\hat{K}_i-1}^{(i)} < 0, \end{cases}$$

and, for $k = 2, \ldots, \hat{K}_i - 1$,

$$\hat{c}_k^{(i)} = \begin{cases} \frac{2\lambda_2}{\hat{L}_k^{(i)}}, & \text{if } \hat{\mu}_k^{(i)} - \hat{\mu}_{k-1}^{(i)} < 0, \hat{\mu}_{k+1}^{(i)} - \hat{\mu}_k^{(i)} > 0, \\ -\frac{2\lambda_2}{\hat{L}_k^{(i)}}, & \text{if } \hat{\mu}_k^{(i)} - \hat{\mu}_{k-1}^{(i)} > 0, \hat{\mu}_{k+1}^{(i)} - \hat{\mu}_k^{(i)} < 0, \\ 0, & \text{if } (\hat{\mu}_k^{(i)} - \hat{\mu}_{k-1}^{(i)})(\hat{\mu}_{k+1}^{(i)} - \hat{\mu}_k^{(i)}) > 0. \end{cases}$$

The result implies that the sample mean (as an unbiased estimate of true mean) of a local minimum/maximum segment (except it is located at either end) is shifted towards 0 due to fused-lasso penalty. The bias is positively proportional to λ_2 and negatively proportional to the length of the segment. It is more important to notice that there exists no configuration where a local minimum/maximum segment has a jump size (relative to neighboring segments) less than the amount of bias. It means that a CNV with small jump size or small length could possibly be merged into neighboring segments, if λ_2 is set too large.

Bias induced by group-fused-lasso penalty

The subgradient for group-fused-lasso penalty is given in the following Proposition 1.

Proposition 1: The β_{ij}'s involved in group-fused-lasso penalty have subgradient given by

$$s_{ij}^{(3)} = \begin{cases} -e_{i2}, & \text{if } j = 1, \\ e_{ij} - e_{i,j+1}, & \text{if } 1 < j < N, \\ e_{iN}, & \text{if } j = N, \end{cases} \tag{S.4}$$

for $i = 1, \ldots, M$, where $\mathbf{e}_j = (e_{1j}, \ldots, e_{Mj})^T$ for $j = 2, \ldots, M$ are given by

$$\mathbf{e}_j = \begin{cases} \left(\frac{\beta_{1j}-\beta_{1,j-1}}{||\boldsymbol{\beta}_{(j)}-\boldsymbol{\beta}_{(j-1)}||_{\ell_2}}, \ldots, \frac{\beta_{Mj}-\beta_{M,j-1}}{||\boldsymbol{\beta}_{(j)}-\boldsymbol{\beta}_{(j-1)}||_{\ell_2}}\right)^T, & \text{if } ||\boldsymbol{\beta}_{(j)} - \boldsymbol{\beta}_{(j-1)}||_{\ell_2} > 0, \\ \text{any } (e_{1j}, \ldots, e_{Mj})^T \text{ s.t. } ||\mathbf{e}_j||_{\ell_2} \le 1, & \text{if } ||\boldsymbol{\beta}_{(j)} - \boldsymbol{\beta}_{(j-1)}||_{\ell_2} = 0. \end{cases} \tag{S.5}$$

Proof: The proof follows a similar technique used in the proof of Lamma A.1 in [3]. Let $\mathbf{T} = [-\mathbf{I}_M, \mathbf{I}_M]$, where $\mathbf{I}_M$ is $M \times M$ identity matrix. Then, for any $2 \le j \le N$,

$$h(\boldsymbol{\beta}_{(j-1)}, \boldsymbol{\beta}_{(j)}) \triangleq ||\boldsymbol{\beta}_{(j)} - \boldsymbol{\beta}_{(j-1)}||_{\ell_2} = ||\mathbf{T}[\boldsymbol{\beta}_{(j-1)}^T, \boldsymbol{\beta}_{(j)}^T]^T||_{\ell_2}.$$

For the j such that $||\boldsymbol{\beta}_{(j)} - \boldsymbol{\beta}_{(j-1)}||_{\ell_2} > 0$, the sub-gradient is reduced to regular gradient, and thus can be derived in a usual way. We now focus on the j such that $||\boldsymbol{\beta}_{(j)} - \boldsymbol{\beta}_{(j-1)}||_{\ell_2} = 0$, i.e., the subgradient of β_{ij} at 0. By Cauchy-Schwartz inequality, we have

$$\begin{aligned} h(\boldsymbol{\beta}_{(j-1)}, \boldsymbol{\beta}_{(j)}) &\ge ||\mathbf{T}[\boldsymbol{\beta}_{(j-1)}^T, \boldsymbol{\beta}_{(j)}^T]^T||_{\ell_2} ||\mathbf{e}_j||_{\ell_2} \\ &\ge \, < \mathbf{T}[\boldsymbol{\beta}_{(j-1)}^T, \boldsymbol{\beta}_{(j)}^T]^T, \mathbf{e}_j > \\ &= h(\mathbf{0}) + < [\boldsymbol{\beta}_{(j-1)}^T, \boldsymbol{\beta}_{(j)}^T]^T - \mathbf{0}, \mathbf{T}^T\mathbf{e}_j > \end{aligned}$$

where $\mathbf{e}_j$ is any vector such that $||\mathbf{e}_j||_{\ell_2} \le 1$. It follows by the definition of subgradient that $\mathbf{T}^T\mathbf{e}_j = [-\mathbf{e}_j^T, \mathbf{e}_j^T]^T$ is the subgradient for $[\boldsymbol{\beta}_{(j-1)}^T, \boldsymbol{\beta}_{(j)}^T]^T$. $\square$

The bias induced by the group-fused-lasso penalty can be derived from the analytic form of subgradient accordingly and is given in the following Proposition 2.

Proposition 2: In model (S.1) with $\lambda_1 = 0$ and $\lambda_2 = 0$, the fitted means of segments for sequence i can be expressed as

$$\hat{\mu}_k^{(i)} = \frac{1}{\hat{L}_k^{(i)}} \sum_{j \in \hat{R}_k^{(i)}} x_{ij} + \hat{c}_k^{(i)}, \quad k = 1, \ldots, \hat{K}_i,$$

where

$$\hat{c}_k^{(i)} = \begin{cases} \frac{\lambda_3}{\hat{L}_1^{(i)}} \cdot r_i(\hat{j}_1^{(i)}), & \text{if } k = 1, \\ -\frac{\lambda_3}{\hat{L}_k^{(i)}} \cdot \left[r_i(\hat{j}_{k-1}^{(i)}) - r_i(\hat{j}_k^{(i)}) \right], & \text{if } 2 \le k \le \hat{K}_i - 1, \\ -\frac{\lambda_3}{\hat{L}_{\hat{K}_i}^{(i)}} \cdot r_i(\hat{j}_{\hat{K}_i - 1}^{(i)}), & \text{if } k = \hat{K}_i, \end{cases}$$

and

$$r_i(j) \triangleq \frac{\hat{\beta}_{ij} - \hat{\beta}_{i,j-1}}{||\hat{\boldsymbol{\beta}}_{(j)} - \hat{\boldsymbol{\beta}}_{(j-1)}||_{\ell_2}}.$$

Proof: The proof follows a similar technique used in the proof of Lemma 2.1 in [3]. Following the subgradient condition (S.2) in case $\lambda_1 = 0$ and $\lambda_2 = 0$, we have

$$\hat{\mu}_k^{(i)} = \frac{1}{\hat{L}_k^{(i)}} \sum_{j \in \hat{R}_k^{(i)}} \hat{\beta}_{ij} = \frac{1}{\hat{L}_k^{(i)}} \sum_{j \in \hat{R}_k^{(i)}} x_{ij} - \frac{\lambda_3}{\hat{L}_k^{(i)}} \sum_{j \in \hat{R}_k^{(i)}} s_{ij}^{(3)}.$$

By Proposition 1 and simple algebra, we have

$$\sum_{j \in \hat{R}_k^{(i)}} s_{ij}^{(3)} = \begin{cases} -e_{i,\hat{j}_1^{(i)}}, & \text{if } k = 1, \\ e_{i,\hat{j}_{k-1}^{(i)}} - e_{i,\hat{j}_k^{(i)}}, & \text{if } 2 \le k \le \hat{K}_i - 1, \\ e_{i,\hat{j}_{\hat{K}_i-1}^{(i)}}, & \text{if } k = \hat{K}_i. \end{cases}$$

Note that at jump points, subgradient has explicit form as shown in Proposition 1. It follows that $e_{i,\hat{j}_k^{(i)}} = r_i(\hat{j}_k^{(i)})$, for $k = 1, \ldots, \hat{K}_i - 1$, where $r_i(\cdot)$ is defined in Proposition 2. □

Some interesting implications follow immediately. For sequence i, consider one of its fitted segment k with end points $[\hat{j}_{k-1}^{(i)}, \hat{j}_k^{(i)} - 1]$. If no other sequences share change points at these two ends, then the bias term $\hat{c}_k^{(i)}$ reduces to what it appears in model (S.1) with fused-lasso term only ($\lambda_1 = 0$ and $\lambda_3 = 0$). If m out of M sequences share change points at these two ends and also assume the jump size at these two locations for all the m sequences are roughly the same, then the absolute value of the bias term can be approximately written as $\frac{2\lambda_3}{\hat{L}_k^{(i)}} \cdot \frac{1}{\sqrt{m}}$. It means that if more than one sequences share change points at the same coordinate, then they can benefit from each other to reduce their individual bias, relative to the bias induced by fused-lasso penalty specific to each individual sequence.

Asymptotic behavior

Now we try to give a justification of the order of the magnitude of λ_2 and λ_3 in compatible with their large sample behavior, say, as $N \to \infty$. When the number of sequences M in segmentation task is relatively large, extra caution is needed for λ_3. Again, we discuss asymptotic behavior of the solution influenced by fused-lasso and group-fused-lasso separately for easier exhibition.

Asymptotic behavior for fused-lasso penalty

In fused-lasso model ($\lambda_1 = 0$ and $\lambda_3 = 0$), the justification is directly inspired by the proof of Theorem 2.3 in [3]. Denote the event

$$\mathcal{E}_i = \{\hat{\mathcal{J}}_i = \mathcal{J}_i\} \cap \{\text{sign}(\hat{\beta}_{ij} - \hat{\beta}_{i,j-1}) = \text{sign}(\beta_{ij} - \beta_{i,j-1}), \forall j \in \mathcal{J}_i\},$$

for $i = 1, \ldots, M$ respectively. This event means that all jump points and the direction of jumps are correctly identified for each sequence i. A necessary condition required for λ_2 is summarized in Proposition 3.

Proposition 3: It is required that $\lambda_2 = O(\sqrt{\log N})$ to ensure $\lim_{N\to\infty} \mathbb{P}(\mathcal{E}_i) = 1$ for $i = 1, \ldots, M$, at the linear rate.

This asymptotic behavior follows directly the proof of Theorem 2.3 in [3]. We have some quick remarks:

1) If the signal of each sequence is not normalized, then $\lambda_{2,i} = c_2\sigma_i\sqrt{\log N}$, specific to sequence i.

2) In order to ascertain a CNV segment with length L and jump size δ, the bias needs to satisfy $\frac{2\lambda_{2,i}}{L} = \frac{2c_2\hat{\sigma}_i\sqrt{\log N}}{L} < \delta$, i.e., $c_2 < \frac{1}{2\sqrt{\log N}} \cdot \frac{\delta}{\sigma_i}L$. Here, $\frac{\delta}{\sigma_i}$ can be interpreted as signal-to-noise ratio (SNR). For a specific platform, one may get a sense of the magnitude of SNR and L from prior knowledge. In practice, it is desired to take as large value of c_2 as possible to ensure the sparsity of the segmentation, but not too large in order to compensate for the constraint of signal strength ($\frac{\delta}{\sigma_i}L$). Based on our experiences of analysis of Illumina data [5], the results are not sensitive to the choice of c_2, provided that it falls into a reasonable range.

Asymptotic behavior for group-fused-lasso penalty

In group-fused-lasso model ($\lambda_1 = 0$ and $\lambda_2 = 0$), we have similar requirement of λ_3 as for λ_2, which is given in Proposition 4.

Proposition 4: It is required that $\lambda_3 = O(\sqrt{M}\sqrt{\log N})$ to ensure $\lim_{N\to\infty}\mathbb{P}(\cap_{i=1}^{M}\mathcal{E}_i) = 1$, at the linear rate.

Proof: For simplicity, we prove under the condition that ϵ_{ij} are i.i.d. $\mathcal{N}(0,1)$ (after σ_i is normalized to 1), while this condition can be relaxed [3]. We also follow the same technique used in the proof of Theorem 2.3 in [3]. Let $d_{ij} = \beta_{ij} - \beta_{i,j-1}$, $\hat{d}_{ij} = \hat{\beta}_{ij} - \hat{\beta}_{i,j-1}$, and $d_{ij}^{\epsilon} = \epsilon_{ij} - \epsilon_{i,j-1}$. Also denote $\mathbf{d}_j^{\epsilon} = (d_{1j}^{\epsilon}, \ldots, d_{Mj}^{\epsilon})^T$ and $\mathcal{J} = \cup_{i=1}^{M}\mathcal{J}_i$. By the subgradient condition (S.2), for each i, $\mathcal{E}_i$ holds if and only if

$$d_{ij}^{\epsilon} = \lambda_3[2e_{ij} - e_{i,j-1} - e_{i,j+1})], \quad \text{for } j \in \mathcal{J}_i^c, \tag{S.6}$$

and

$$|\hat{d}_{ij}| > 0, \quad \text{for } j \in \mathcal{J}_i. \tag{S.7}$$

Condition (S.7) has direct relevance to the bias issue, as discussed above. Now we focus on condition (S.6), which implies that

$$\max_{j \in \mathcal{J}^c} ||\mathbf{d}_j^\epsilon||_{\ell_2} = \max_{j \in \mathcal{J}^c} \lambda_3 ||2\mathbf{e}_j - \mathbf{e}_{j-1} - \mathbf{e}_{j+1}||_{\ell_2} < 4\lambda_3.$$

It is left to show that $\mathbb{P}(\max_{j \in \mathcal{J}^c} ||\mathbf{d}_j^\epsilon||_{\ell_2} \geq 4\lambda_3) = \mathbb{P}(\max_{j \in \mathcal{J}^c} ||\mathbf{d}_j^\epsilon/\sqrt{2}||_{\ell_2}^2 \geq 8\lambda_3^2) \to 0$ as $N \to \infty$ for $i = 1, \ldots, M$. Note that for each j, $d_{1j}^\epsilon, \ldots, d_{Mj}$ are i.i.d. $\mathcal{N}(0, 2)$, so $||\mathbf{d}_j^\epsilon/\sqrt{2}||_{\ell_2}^2 \sim \chi_M^2$. Then we have

$$\begin{aligned}
& \mathbb{P}(\max_{j \in \mathcal{J}^c} ||\mathbf{d}_j^\epsilon/\sqrt{2}||_{\ell_2}^2 \geq 8\lambda_3^2) \\
= \; & \mathbb{P}(\cup_{j \in \mathcal{J}^c} ||\mathbf{d}_j^\epsilon/\sqrt{2}||_{\ell_2}^2 \geq 8\lambda_3^2) \\
\leq \; & \sum_{j \in \mathcal{J}^c} \mathbb{P}(||\mathbf{d}_j^\epsilon/\sqrt{2}||_{\ell_2}^2 \geq 8\lambda_3^2) \\
= \; & |\mathcal{J}^c| \mathbb{P}(||\mathbf{d}_j^\epsilon/\sqrt{2}||_{\ell_2}^2 \geq 8\lambda_3^2) \\
\leq \; & \exp\left[-\frac{1}{2}(8\lambda_3^2 - M) + \log |\mathcal{J}^c| - \frac{M}{2} \log \frac{M}{8\lambda_3^2} \right].
\end{aligned}$$

Here the first inequality is due to union bound and the second inequality is due to Chernoff's bound for χ_M^2 distribution. Under the assumption on sparsity of the change points, we have $|\mathcal{J}^c| = O(N)$ for fixed M. In our settings, M is fixed (which may rise up to thousands) while $N \to \infty$, yet in practice, M is not negligible with respect to $\sqrt{\log N}$. For example, $\sqrt{\log(10^6)} \approx 3.72$, and it is not uncommon to have more than 4 sequences for joint segmentation. Therefore, it is necessary to have $\lambda_3 = O(\sqrt{M}\sqrt{\log N})$. $\square$

We also have some remarks on how to determine λ_3:

1) If the signal of each sequence is not normalized, then $\lambda_{3,i} = c_3 \sigma_i \sqrt{pM}\sqrt{\log N}$. The choice of p is decided case by case and discussed in the main text.

2) Following the above discussion about bias induced by group-fused-lasso penalty, if m out of M sequences carry CNVs with exactly the same boundary, the bias can be approximately written as $\frac{2c_3\sigma_i\sqrt{\log N}}{\hat{L}_k^{(i)}} \cdot \frac{\sqrt{pM}}{\sqrt{m}}$. On one hand, if p is over estimated so that pM is much larger than m, the model would be over penalized and introduce more bias than that is attributed to individual fused-lasso penalty, and thus does not benefit from joint analysis; on the other hand, if pM is set too small, we have insufficient control on the sparsity of each sequence, so that it has to be compensated by the fused-lasso penalty. This is the reason why we need to incooperate $\rho(p)$ to re-weight the relative influence of the two penalties.

Details in calling procedure

We specify the likelihood functions of LRR and BAF signals in the log-likelihood ratio (8) in the main text as follows. For BAF signal, the likelihood is usually modeled for different copy number states as a mixture of densities surrounding a few possible BAF values corresponding to different genotypes [1, 4]. When population frequencies for allele A and B, p_A and p_B, are available or can be estimated from data, we have

$$L_{\text{BAF}}(x; c) = \sum_{s=0}^{c} \binom{c}{s} p_A^{c-s} p_B^{s} \phi_s(x; \mu_s, \sigma_s^2), \quad \text{for } c = 0, 1, 2, 3, 4.$$

where $\phi_s(\cdot; \mu_s, \sigma_s^2)$ is normal density for state s. The details in model and parameter specification are listed in Supplementary Table 1.

In case where population frequencies p_A and p_B are not available, we might use an alternative likelihood function for BAF [5], defined by

$$L_{\text{BAF}}(x; c) = \max_{s \in \{0, \ldots, c\}} \phi_s(x; \mu_s, \sigma_s^2), \quad \text{for } c = 0, 1, 2, 3, 4,$$

where all parameters are defined in the same way (see Supplementary Table 1).

Supplementary Table 1: Model and parameter specification in BAF signal for each copy number state. $\hat{\sigma}_x$ is empirically estimated from BAF values in $(0.4, 0.6)$ for each individual.

c	s	Genotype	$\phi_s(\cdot)$	μ_s	σ_s
0	0	Null	normal	$1/2$	$10\hat{\sigma}_x$
1	0, 1	A, B	half normal	$0, 1$	$\hat{\sigma}_x$
2	0, 2	AA, BB	half normal	$0, 1$	$\hat{\sigma}_x$
	1	AB	normal	$1/2$	$\hat{\sigma}_x$
3	0, 3	AAA, BBB	half normal	$0, 1$	$\hat{\sigma}_x$
	1, 2	AAB, ABB	normal	$1/3, 2/3$	$\hat{\sigma}_x$
4	0, 4	AAAA, BBBB	half normal	$0, 1$	$\hat{\sigma}_x$
	1, 2, 3	AAAB, AABB, ABBB	normal	$1/4, 1/2, 3/4$	$\hat{\sigma}_x$

For LRR signal, the likelihood function is simply defined by normal density:

$$L_{\text{LRR}}(y; c) = \phi(y; \mu_c, \sigma_c^2).$$

For $c = 0, 1, 3, 4$, μ_c and σ_c^2 are estimated based on the data $\mathbf{y}_R$ in segment R being considered, while μ_2 and σ_2^2 are estimated from the data of the whole chromosome on which segment R locates or, locally, from the data of a few hundred markers flanking the segment.

References

[1] S. Colella, C. Yau, J. M. Taylor, G. Mirza, H. Butler, P. Clouston, A. S. Bassett, A. Seller, C. C. Holmes, and J. Ragoussis. Quantisnp: An objective bayes hidden-markov model to detect and accurately map copy number variation using snp genotyping data. *Nucleic Acids Research*, 35:2013–2025, 2007.

[2] J. Friedman, T. Hastie, H. Höfling, and R. Tibshirani. Pathwise coordinate optimization. *The Annals of Applied Statistics*, 1:302–332, 2007.

[3] A. Rinaldo. Properties and refinements of the fused lasso. *The Annals of Statistics*, 37:2922–2952, 2009.

[4] K. Wang, M. Li, D. Hadley, R. Liu, J. Glessner, S. F. A. Grant, H. Hakonarson, and M. Bucan. Penncnv: An integrated hidden markov model designed for high-resolution copy number variation detection in whole-genome snp genotyping data. *Genome Research*, 17:1665–1674, 2007.

[5] Z. Zhang, K. Lange, R. Ophoff, and C. Sabatti. Reconstructing dna copy number by penalized estimation and imputation. *The Annals of Applied Statistics*, 4:1749–1773, 2010.
