# Supplementary material for: Reconstructing DNA copy number by joint segmentation of multiple sequences
Source: BMC Bioinformatics. 2012 Aug 16;13:205. doi: 10.1186/1471-2105-13-205 (PMC3534631; doi:10.1186/1471-2105-13-205)
Supplement: Additional file 2 — Table S1. Regions of allelic imbalance imputed to the HapMap sample NA06991. [file 1471-2105-13-205-S2.pdf]

**Table S1. Regions of allelic imbalance imposed to the HapMap sample NA06991[a]**

| Region | Aberration Type[b] | Chr[c] | bp Start[c] | bp End[c] | #SNP[d] | #hetSNP[d] |
|---|---|---|---|---|---|---|
| 1 | CN-LOH | 5 | 1 | 47700000 | 9397 | 2756 |
| 2 | Loss | 5 | 111789971 | 112521346 | 156 | 79 |
| 3 | Gain | 8 | 1 | 45200000 | 12564 | 3830 |
| 4 | Gain | 8 | 128432670 | 129207869 | 218 | 91 |
| 5 | Loss | 9 | 1 | 50600000 | 11201 | 3889 |
| 6 | Loss | 10 | 84504379 | 94825178 | 1988 | 648 |
| 7 | Gain | 12 | 1 | 132449811 | 27131 | 8818 |
| 8 | Loss | 13 | 31766569 | 31892852 | 37 | 10 |
| 9 | CN-LOH | 17 | 7431864 | 11747138 | 1150 | 308 |
| 10 | CN-LOH | 17 | 22300000 | 78774742 | 9713 | 3205 |
| Total number of modified heterozygous SNPs | | | | | | 23634 |
| Total number of heterozygous SNPs on autosome | | | | | | 176207 |
| Total number of SNPs on autosome | | | | | | 547359 |

a: For more details, see Staaf J, et al. (2008) Genome Biology 9: R136.

b: Aberration type includes one-copy gain/loss and copy-neutral loss of heterozygosity (CN-LOH).

c: Chromosome and base pair start/end position where each simulated region is located.

d: Listed is the number of SNPs and those with heterozygous genotypes within each region.
