# Supplementary material for: Reconstructing DNA copy number by joint segmentation of multiple sequences
Source: BMC Bioinformatics. 2012 Aug 16;13:205. doi: 10.1186/1471-2105-13-205 (PMC3534631; doi:10.1186/1471-2105-13-205)
Supplement: Additional file 3 — Table S2. Speed comparison of three methods: GFL, BAFsegmentation and PSCN. [file 1471-2105-13-205-S3.pdf]

Table S2. Speed comparison of three methods: GFL, BAFsegmentation and PSCN

Method	Time (sec.)[a]
GFL	21.97 (1.31)
BAFsegmentation	41.73 (-)
PSCN	1154.18 (74.73)

a: The average time (standard deviation) required for analysis of one sample (in second).
