# Supplementary material for: Reconstructing DNA copy number by joint segmentation of multiple sequences
Source: BMC Bioinformatics. 2012 Aug 16;13:205. doi: 10.1186/1471-2105-13-205 (PMC3534631; doi:10.1186/1471-2105-13-205)
Supplement: Additional file 4 — Table S3. Sample information and reference CNV regions summarized for each sample by their types and sizes. [file 1471-2105-13-205-S4.pdf]

Table S3. Sample information and reference CNV regions summarized for each sample by their types and sizes

Sample	Gender	Ancestry	Resource	Type	<10k	10−50k	50−100k	>100k	Total
NA15510	Female	European[a]	PDR[b]	loss	12	25	3	7	47
				gain	0	0	1	4	5
				total	12	25	4	11	52
NA18517	Female	YRI	HapMap	loss	10	22	4	4	40
				gain	1	3	1	8	13
				total	11	25	5	12	53
NA18576	Female	CHB	HapMap	loss	13	16	4	5	38
				gain	0	2	2	4	8
				total	13	18	6	9	46
NA18980	Female	JPT	HapMap	loss	8	16	1	4	29
				gain	0	0	1	3	4
				total	8	16	2	7	33

a: The ancestry of NA15510 was not recorded but inferred in Korbel JO et al. (2007) Science 318: 420-426.
b: Abbreviation: PDR = Polymorphism Discovery Resource.
