# Supplementary material for: Reconstructing DNA copy number by joint segmentation of multiple sequences
Source: BMC Bioinformatics. 2012 Aug 16;13:205. doi: 10.1186/1471-2105-13-205 (PMC3534631; doi:10.1186/1471-2105-13-205)
Supplement: Additional file 5 — Table S4. Summary of results for four real samples under different CNV analyses. [file 1471-2105-13-205-S5.pdf]

Table S4. Summary of results for four real samples under different CNV analyses

Analysis	ρ^a	M^a	NA15510 #Det.[b]	NA15510 #Ovlp.[b]	NA18517 #Det.	NA18517 #Ovlp.	NA18576 #Det.	NA18576 #Ovlp.	NA18980 #Det.	NA18980 #Ovlp.	Time (min.)[c]
Analysis A: GFL done on averaged signal for each platform[e]											
O1Q[d]	1	1	92	34	73	22	71	21	69	20	0.3
O2Q[d]	1	1	114	22	92	24	111	15	95	11	0.9
Union[d]	-	-	170	38	144	34	160	25	145	22	1.2
Analysis B: GFL done on averaged signal of both platforms jointly											
	0	2	128	40	108	33	96	21	104	23	4.2
Analysis C: GFL done on three replicates separately for each platform[e]											
O1Q	1	1	66	31	65	22	43	19	48	15	0.9
O2Q	1	1	68	23	65	22	65	12	59	13	2.8
Union	-	-	102	36	109	33	93	25	91	20	3.7
Analysis D: GFL done on three replicates jointly for each platform											
O1Q	0	3	64	32	66	22	54	21	53	18	1.1
O2Q	0	3	75	22	70	24	65	11	49	12	3.1
Union	-	-	106	36	115	33	96	22	83	21	4.2
Analysis E: GFL done on three replicates of both platforms jointly[e]											
	0	6	80	38	82	32	69	25	56	15	8.5
MPCBS: Segmentation done on three replicates of both platforms jointly											
	-	-	98	34	88	28	59	18	68	21	313.9

a: Tuning parameters used in segmentation: $c_1 = 0.1$, $c_2 = 2$, $c_3 = 2$ and $p = 1$; ρ and M are specified for each analysis.
b: The number of CNVs detected (Det.) and overlapping (Ovlp.) with reference results.
c: Average computation time (in minute) for four samples under different analyses.
d: In Analysis A, C and E, GFL is first applied on data from Illumina Omni1-Quad (O1Q) and Omni2.5-Quad (O2Q) respectively. Results from two platforms are combined by taking union of them.
e: Analysis A, C and E correspond to Analysis 1, 2 and 3 respectively in the main text.
