# Supplementary figures and images for: Reconstructing DNA copy number by joint segmentation of multiple sequences
Source: BMC Bioinformatics. 2012 Aug 16;13:205. doi: 10.1186/1471-2105-13-205 (PMC3534631; doi:10.1186/1471-2105-13-205)

# Chromosome 8

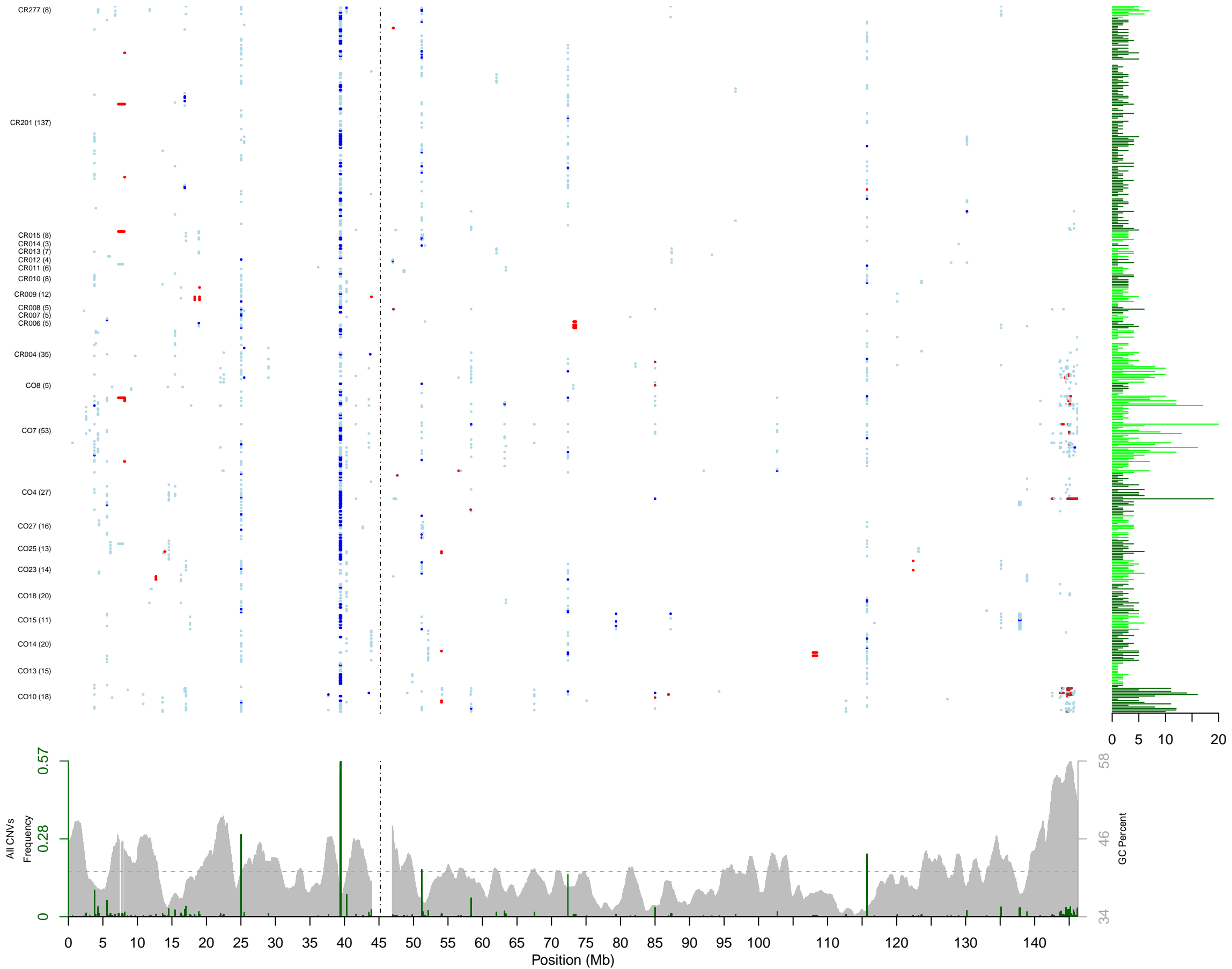

Supplement: Additional file 6 — Figure S1. Visualization of pedigree-wise CNV analysis results of Chromosome 8 data in the bipolar disorder study. In the main body of the plot, CNVs estimated for each individual are marked by small segments with color code: CN= 0 in blue, CN=1 in light blue, CN=3 in red and CN=4 in brown. Each subject is a row, each SNP a column. Subjects belonging to the same pedigree are stacked together. The pedigree names are indicated on the left-hand side with the number of pedigree members included in parentheses. On the right-hand side, the barplot represents the number of CNVs detected per subject. Two shades of green are switched alternately to indicate the pedigree to which the subject belongs. At the bottom, the gray histogram shows the GC content along the chromosome. Coordinated with the representation of CNVs in the main body, the green histogram counts the frequency of CNVs among the subjects represented. Vertical dotted line marks the centromere. [file 1471-2105-13-205-S6.pdf]
